# Enhancing Antifungal Treatment of *Candida albicans* with Hypericin-Loaded Nanostructured Lipid Carriers in Hydrogels: Characterization, In Vitro, and In Vivo Photodynamic Evaluation

**DOI:** 10.3390/ph16081094

**Published:** 2023-08-01

**Authors:** Mariana Rillo Sato, João Augusto Oshiro-Junior, Camila Fernanda Rodero, Fernanda Isadora Boni, Victor Hugo Sousa Araújo, Taís Maria Bauab, Dean Nicholas, John Francis Callan, Marlus Chorilli

**Affiliations:** 1 School of Pharmaceutical Sciences, São Paulo State University (UNESP), Araraquara 14800-903, SP, Brazil; mari_sato_@hotmail.com (M.R.S.); camilafrodero@hotmail.com (C.F.R.); boni.fernanda@gmail.com (F.I.B.); victorhunterhsa@hotmail.com (V.H.S.A.); tais.bauab@unesp.br (T.M.B.); 2 Graduation Program in Pharmaceutical Sciences, State University of Paraíba, Campina Grande 58429-500, PB, Brazil; joaooshiro@yahoo.com.br; 3 Biomedical Sciences Research Institute, University of Ulster, Coleraine BT52 1SA, UK; nicholas-d@ulster.ac.uk (D.N.); j.callan@ulster.ac.uk (J.F.C.)

**Keywords:** mucoadhesiveness, nanostructured system, photosensitizer, yeast, antifungal agent

## Abstract

Background: Vulvovaginal candidiasis (VVC) is a worldwide public health problem caused predominantly by the opportunistic polymorphic fungus *Candida albicans*, whose pathogenicity is associated with its morphological adaptability. To potentiate the treatment of *C. albicans*-induced VVC by an alternative method as photodynamic therapy (PDT), hypericin (Hy), a potent photosensitizer compound was incorporated into a nanostructured lipid carrier (NLC) and dispersed in hydrogel (HG). Methods: After preparation of the sonication process, an NLC loaded with Hy was dispersed in HG based on Poloxamer 407 and chitosan obtaining Hy.NLC-HG. This hydrogel system was physically and chemically characterized and its in vitro and in vivo photodynamic and antifungal effects were evaluated. Results: Through scanning electron microscopy, it was possible to observe a hydrogel system with a porous polymeric matrix and irregular microcavities. The Hy.NLC-HG system showed mucoadhesive properties (0.45 ± 0.08 N) and a satisfactory injectability (15.74 ± 4.75 N.mm), which indicates that it can be easily applied in the vaginal canal, in addition to a controlled and sustained Hy release profile from the NLC-HG of 28.55 ± 0.15% after 720 min. The in vitro antibiofilm assay significantly reduced the viability of *C. albicans* (*p* < 0.001) by 1.2 log_10_ for Hy.NLC-HG/PDT and 1.9 log_10_ for PS/PDT, Hy.NLC/PDT, and free RB/PDT, compared to the PBS/PDT negative control. The in vivo antifungal evaluation showed that animals treated with the vaginal cream (non-PDT) and the PDT-mediated Hy.NLC-HG system showed a significant difference of *p* < 0.001 in the number of *C. albicans* colonies (log) in the vaginal canal, compared to the inoculation control group. Conclusions: Thus, we demonstrate the pharmaceutical, antifungal, and photodynamic potential of hydrogel systems for Hy vaginal administration.

## 1. Introduction

Vulvovaginal candidiasis (VVC) is a common infection in the female genital tract and is predominantly caused (77–95% of cases) by the opportunistic polymorphic fungus *Candida albicans* [1]. Due to its commonality, VVC represents a considerable global public health concern. *C. albicans*’ high prevalence can be attributed to its morphological adaptability, enabling adhesion to the mucosal surface in yeast or filamentous (pseudohyphae or hyphae) forms, which can potentially cause damage via hydrolytic enzyme production [2].

Given this scenario, the search for effective alternative topical treatments has been intensified [3]. Photodynamic therapy (PDT) has emerged as a promising approach in managing bacterial and fungal infections, mainly due to its multi-targeted action mechanism that results in microbial photoinactivation [4]. For PDT to effectively combat these pathogens, a non-toxic photosensitizer (PS) is crucial. Hypericin (Hy), a promising compound derived from St. John’s wort, triggers catalytic reactions when exposed to light, leading to the generation of reactive oxygen species that damage intracellular molecules [5,6]. However, Hy’s lipophilic nature and its water-insolubility, as well as its insolubility in most non-polar solvents [7], could potentially hamper the effectiveness of this photosensitizer in VVC therapy. These complications underscore the need for innovative therapeutic and nanotechnological strategies.

Nanostructured lipid carriers (NLCs) have notably piqued the interest of the scientific community. Created from a blend of liquid and solid lipids at room temperature, NLCs provide an impressive physicochemical stability and effectiveness for integrating specific drugs like Hy into an imperfect lipid matrix composed of disordered crystalline structures [8].

Conversely, hydrogels (HGs) are three-dimensional polymeric networks established by macromolecules interconnected via physical interactions (polymeric chain entanglements) or chemical interactions (covalent-crosslinked bonds). These structures possess several advantages, including biocompatibility, high porosity, and the ability to swell in contact with water [9,10] making them ideal for drug and nanostructure incorporation.

Given these considerations, NLCs loaded with Hy and dispersed in HG could potentially extend the treatment’s residence time, thereby decreasing the required dosage and frequency of application. This extension could help prevent or minimize inadvertent removal after on-site administration, reducing patient discomfort during vaginal application and enhancing the acceptability of antifungal therapy [11,12]. Hence, by merging a nanostructured system with a mucoadhesive hydrogel, our work aims to enhance the action of the Hy compound through photodynamic therapy, offering a rapid, safe, and feasible alternative for *C. albicans* treatment.

## 2. Results and Discussion

### 2.1. SEM Microscopy and DLS Analysis

Following the system’s formation, we utilized dynamic light scattering (DLS) to ascertain the hydrodynamic diameter, polydispersity index, and zeta potential of the pure NLC particles. DLS is a widely used nanoparticle characterization technique to measure the intensity of scattered light over time and the effective surface charge on the particles [13]. 

**Figure 1:**
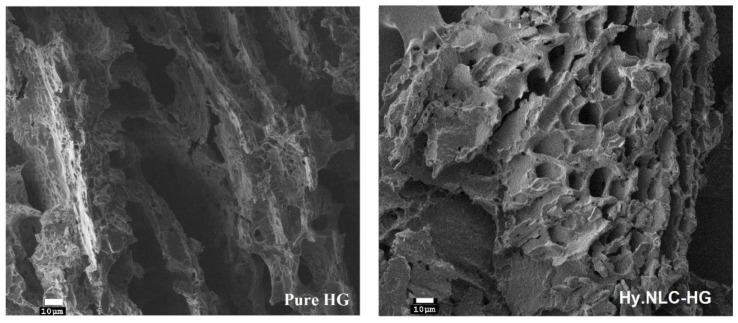
SEM images of the pure HG and Hy.NLC-HG at 500× magnification.

This allowed us to verify the reproducibility of our production process and the physical properties of the developed nanoparticles. Comparatively, our DLS measurements confirmed the homogeneity and physical stability of our system—189.10 ± 2.96 nm, 0.39 ± 0.05 and −31.97 ± 0.21 mV—against Sato et al.’s previous results [14], which displayed values of approximately 142 nm, 0.22, and −20 mV for pure NLCs and 140 nm, 0.27, and −30 mV for Hy-loaded NLCs. We next employed SEM to microscopically evaluate the structural and morphological characteristics of the hydrogel systems, as illustrated in Figure 1.

We determined and reported the mean roughness values (RMS) of the pure HG and the Hy-loaded NLC as 37.4 and 28, respectively. The freeze-dried hydrogels examined displayed a porous network with irregular microcavities. The incorporation of Hy into the NLC and its dispersion in HG resulted in an increased number of pores and diameters within the matrix. This structural modification could potentially expedite the diffusion of biological fluids into the matrix and facilitate the sustained release of Hy. Consequently, this would lead to the expansion of the polymeric mesh, improving the nanocarrier’s diffusion through the hydrogel to reach its target location in the vaginal mucosa.

### 2.2. Mucoadhesion Strength Assay

We assessed the mucoadhesiveness of pure HG and Hy.NLC-HG using the tensile method at 37 ± 0.5 °C with cow vaginal mucosa, which had been previously hydrated in artificial vaginal mucus. The mucoadhesive strength of pure HG was recorded as 0.38 ± 0.04 N. Interestingly, incorporating Hy into the NLC dispersed in HG did not significantly affect (*p* > 0.05) the mucoadhesiveness, which registered at 0.45 ± 0.08 N. The Hy.NLC-HG and mucin mainly interact due to the electrostatic forces between the hydrogel’s cationic amino groups of chitosan and the anionic structures of mucus glycoproteins or mucins in the sialic and sulfonic acid forms [15,16]. The presence of hydrogen bonds between the hydroxyl groups in chitosan and the copolymers of ethoxylated groups of the poloxamer in the hydrogel system also plays a role [17]. Moreover, the hydrogel’s hydration aids in interweaving the polymeric chains with the mucin glycoproteins chains, reinforcing the mucoadhesive process [18]. 

### 2.3. Determination of Syringeability

**Figure 2:**
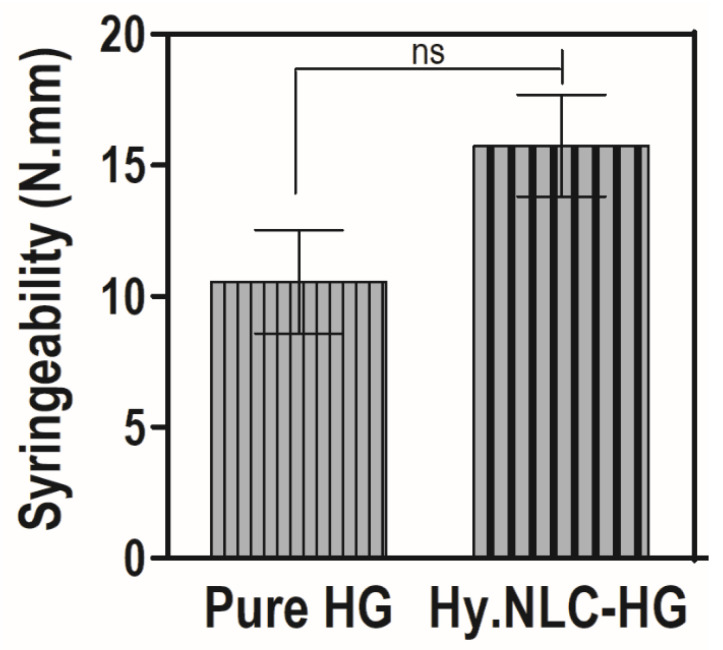
Values of syringeability of pure HG and Hy.NLC-HG. Mean ± SD, *n* = 6. ns = no significant difference (*p* > 0.05).

Syringeability, evaluated using the compression mode, quantifies the required force and work for the system’s application to the vaginal environment through a suitable device, namely a syringe [19]. Figure 2 depicts the syringeability (N.mm) of the pure HG and Hy.NLC-HG.

The hydrogel system showed a favorable syringeability due to the presence of Hy, with a work-required value of 15.74 ± 4.75 N.mm. This did not significantly interfere (*p* > 0.05) with the system’s extrusion compared to the pure HG, which had a value of 10.55 ± 4.83 N.mm. As highlighted in Bruschi et al.’s study [20], syringeability values of less than 20 N.mm could be suitable for vaginal administration due to their application ease. Furthermore, the 3 mL plastic syringe, which was used to simulate a vaginal applicator, has a smaller diameter than that of a vaginal semisolid applicator. Therefore, lower work-required values to expel the formulation from a commercial vaginal applicator might be observed [21]. 

In terms of injectability, the hydrogels were easily injected through a syringe, while their mucoadhesiveness ensured a satisfactory adherence to the vaginal mucosa. Given the anatomical features of the application site, hydrogels can ensure close contact, remaining at the site of infection for a controlled and sustained duration. An increased residence time can help to prevent formulation leakage and reduce the frequency of application [22].

### 2.4. In Vitro Release Assay

**Figure 3:**
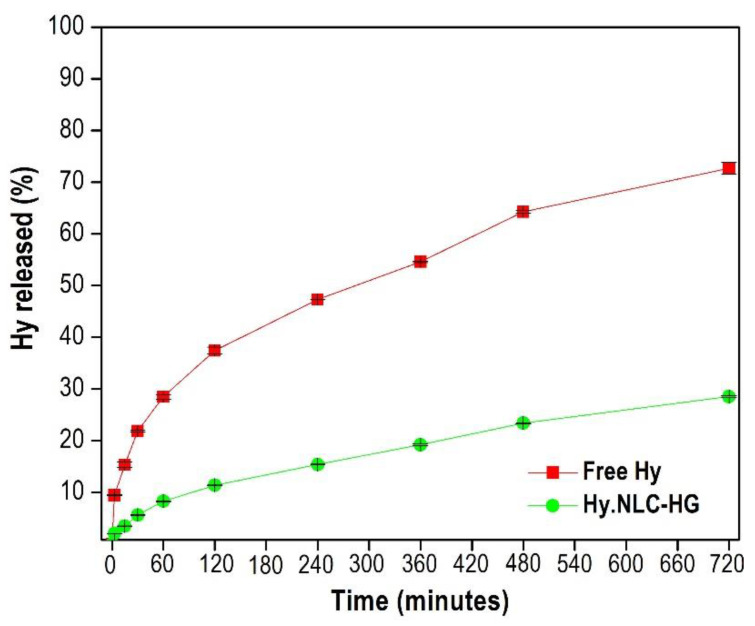
In vitro drug release profiles of free Hy and Hy.NLC-HG. Mean ± SD, *n* = 3. Data were collected at 37 °C ± 0.5 °C.

Phosphate buffer (pH 5) and 3% Tween 80 medium were used to ensure the sink condition for the in vitro release study, conducted for 720 min. This was to simulate the free Hy release profiles and Hy from the NLC dispersed in HG, as illustrated in Figure 3.

Free Hy displayed a controlled release behavior of 21.86 ± 0.23% after 30 min and 72.67 ± 1.13% after 720 min. This trend was observed in the free Hy release assay conducted in the absence of light by Araújo et al. [23] and justified by its attraction to membranes, resulting from an accumulation of saturated lipids. Moreover, studies have reported that after reaching a critical concentration, Hy pigment molecules can form high-molecular-weight and non-fluorescent aggregates in the H-aggregate form [24,25], affecting their release speed. 

In comparison, the system showed a controlled and sustained release from Hy to the release medium of approximately 5.62 ± 0.15% and 28.55 ± 0.15% after 30 and 720 min, respectively, as illustrated in Figure 3. This release profile might be associated with the NLC’s imperfect lipid matrix, which contains structurally disordered crystals. These crystals delay the polymorphic transition and thus minimize the expulsion of Hy [26,27]. The controlled diffusion of Hy might influence its release speed by the hydrogel polymer matrix [28,29]. 

Therefore, the Hy released from the NLC-HG showed a lower ability to cross the synthetic polysulfone membrane. When correlating this behavior to the clinical application, it is assumed that the NLC loaded with hypericin is found in higher concentrations in the vaginal lumen than free Hy. The mucoadhesive hydrogel system could be an effective vehicle for hydrophobic compounds, demonstrating the ability to control Hy release and favor its vaginal administration for localized action.

### 2.5. C. albicans Biofilm Inhibition Assay and Singlet Oxygen Generation Test

**Figure 4:**
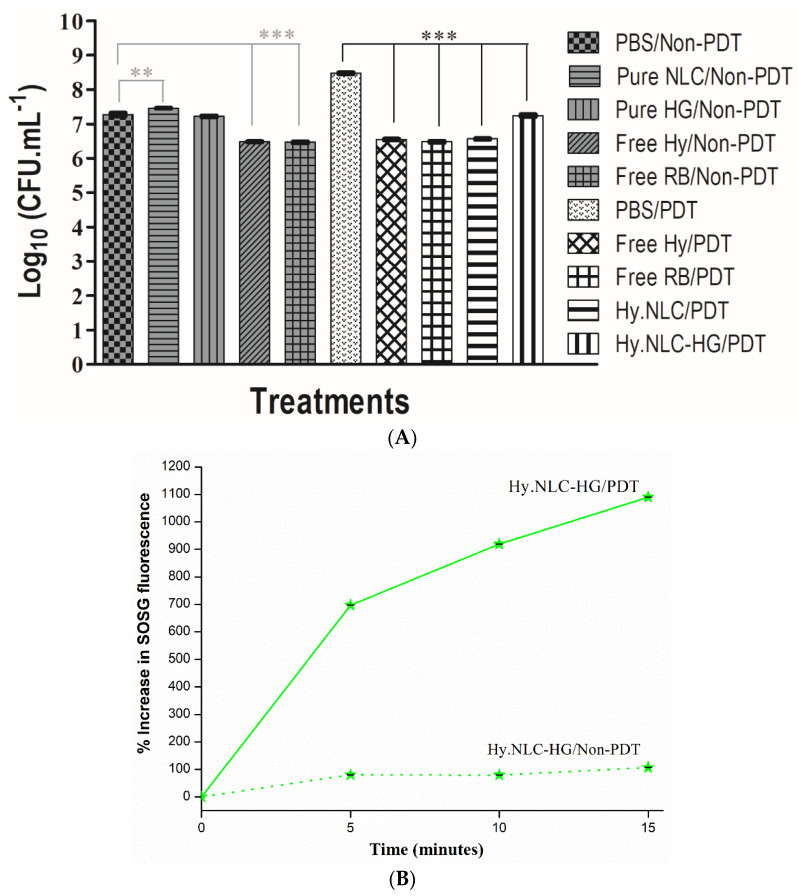
(**A**) Viability count (log_10_) of non-irradiated biofilm cultures (non-PDT) and PDT-treated (LED light at 113 J.cm^−2^, 5 min) by different groups. (**B**) Determination of SOSG fluorescence emission of Hy.NLC-HG. Free RB/non-PDT = positive control, ** *p* < 0.01, *** *p* < 0.001, compared between the treatment and the PBS control groups.

Figure 4A shows the antifungal activity of the groups treated with free photosensitizers and those incorporated into the hydrogel system against *C. albicans* biofilm, and Figure 4B represents the SOSG test of the hydrogel system. Both in vitro assays were performed in the absence of light (non-PDT) and by PDT (LED light at 113 J.cm^−2^).

Biofilms which were not exposed to light sources in the pure NLC, free Hy, and free RB groups demonstrated a significant reduction in *C. albicans* viability compared to the PBS control group. Among these, a notable reduction of approximately 0.8 log_10_ (CFU.mL^−1^) was observed, corresponding to the biofilms treated by the photosensitizers free Hy/non-PDT and free RB/non-PDT (Figure 4A). 

However, a phototoxic effect of the free Hy and free RB solutions, in addition to Hy.NLC and Hy.NLC-HG systems, was observed for biofilm cultures that were exposed to PDT for 5 min after pre-irradiation time (30 min incubation in dark conditions). These groups promoted a significant logarithmic reduction compared to the control group (*p* < 0.001), where the highest mean reductions in the numbers of CFU per milliliter of *C. albicans* were 1.2 log_10_ for Hy.NLC-HG/PDT and 1.9 log_10_ for free Hy/PDT, Hy.NLC/PDT, and free RB/PDT, with efficacies of 90% and 99% loss of viability, respectively [30]. 

Our results are in line with the effects of RB, a xanthene photosensitizer, which has been reported to be effective against various microorganisms, due to its high quantum yield of singlet oxygen generation [31]. The antibiofilm activity of the free Hy, incorporated into NLCs, added into HG and LED-treated, may be explained by the formation of reactive oxygen species that react with several different metabolic pathways [32], as observed by the SOSG test. The chemical reactions between the free Hy/PDT and Hy.NLC/PDT were evidenced in the SOSG study previously conducted by Sato et al. [14], as well as for the Hy.NLC-HG/PDT hydrogel system (Figure 4B) using a probe of the SOSG singlet oxygen. From this indirect measurement, it is estimated that Hy was able to efficiently generate singlet oxygen, as the percentage of the SOSG fluorescence emission gradually increased from 696.58% to 1089.64% during 15 min of irradiation with LED light.

Therefore, even though light activation in Hy did not entirely inhibit the biofilm cultures, they were shown to overcome one of the main resistance mechanisms, the extracellular matrix and the high cell density of the biofilm surrounding the microbial cells, and act as a physical barrier [33,34]. 

### 2.6. Antifungal Evaluation of the Systems in a VVC In Vivo Model

**Figure 5:**
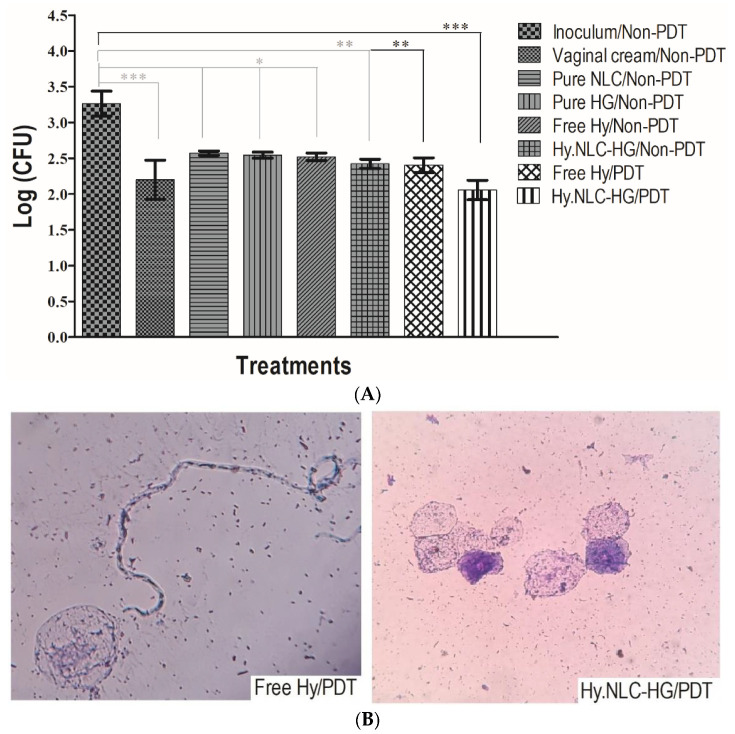
(**A**) *C. albicans* colonies (log) cultivated in the vaginal fluids of female C57BL/6 mice treated in the absence of light (non-PDT) and by PDT (LED light at 113 J.cm^−2^, 5 min). Results are expressed as the mean ± SD of the 4 days of the treatments (1, 3, 5, and 7 days post-inoculation). (**B**) Microscopic images (40× magnification) of *C. albicans* morphology in the vagina of PDT-treated animals. * *p* < 0.05, ** *p* < 0.01, *** *p* < 0.001, compared between the treatment and the inoculum control groups.

To evaluate the antifungal efficacy of Hy against vaginal *C. albicans* infection in vivo, female mice were treated with hydrogel systems in the absence of light (Non-PDT) and by PDT, furthermore, cell morphology in vaginal fluid was analyzed microscopically, as represented in Figure 5A and Figure 5B, respectively.

Figure 5A shows that the *C. albicans* inoculation in the infected group was maintained throughout the assay, as previously determined by Sato et al. [14]. Female mice which were infected and treated in the absence of light with pure systems, NLCs and HG, and Hy in the free state showed a significant difference in the mean number of colonies (*p* < 0.05) over the 4 days of the treatments. However, the difference was significantly improved for animals treated with vaginal cream/non-PDT (*p* < 0.001) compared to the inoculated group, where the mean colony number was 2.20 (log).

Despite the statistically significant log level (2.52) of the free Hy/non-PDT treatment group, the application of an LED to the vaginal canal of the free Hy/PDT-treated animals caused a significant change of *p* < 0.01 (2.40 log) compared to the inoculated group. However, the effectiveness of in vivo PDT was revealed by the intravaginal administration of the Hy.NLC-HG/PDT system, in which the log significance (2.06 CFU) was enhanced to *p* < 0.001, suggesting that Hy in the free form (absent of light and PDT-mediated) and incorporated into the system resulted in antifungal activity against *C. albicans*.

Upon examination of the morphological forms (Figure 5B), a reduction in the growth of *C. albicans* hyphae was observed in the vaginal fluids of animals infected with *C. albicans* and treated with both free Hy/PDT and Hy.NLC-HG/PDT. By the conclusion of the treatment, a limited colonization of elongated hyphae/pseudohyphal forms was evident, which represents an important factor in the virulence of the microorganism, and consequently, a predominant presence of fungal cells in the form of yeast blastospores was noticeable [2]. This observation emphasizes that Hy is likely to be gradually and consistently released from the hydrogel system within the vaginal lumen, aligning with the in vitro release profile (Figure 3). When combined with PDT, it has the potential to significantly contribute to the localized treatment of VCC infection, given that PDT is a relatively non-invasive, cost-effective, safe, and easy procedure [35].

## 3. Materials and Methods

### 3.1. Preparation of the Systems and Dynamic Light Scattering Analysis

The hydrogel (HG) was prepared according to Gratieri et al. [36] with adaptations which involved dispersing 1% (*w*/*w*) of chitosan (Sigma Aldrich^®^, St. Louis, MO, USA) in a 0.5% (*v*/*w*) acetic acid solution under mechanical stirring (model 752, Fisatom^®^, São Paulo, Brazil) at 5000 rpm for 24 h at pH 5. Subsequently, Poloxamer 407 (Sigma Aldrich^®^, St. Louis, MO, USA) 16% (*w*/*w*) was added to the chitosan solution in an ice bath under magnetic stirring at 150 rpm until complete homogenization.

A nanostructured lipid carrier (pure NLC) was sonicated using a sonicator (Qsonica, Q700, Newtown, CT, USA) for a continuous duration of 5 min at 35% amplitude as described by Sato et al. [14]. This process involved the inclusion of an aqueous phase, comprising Poloxamer 407 (3.50% *w*/*v*—Sigma Aldrich^®^, St. Louis, MO, USA) and ultrapure water (Milli-Q Merck Millipore, Burlington, MA, USA), and a lipid phase, which included polyoxyethylene 40 stearate (2.07% *w*/*v*—Sigma Aldrich, St. Louis, MO, USA), capric/caprylic acid triglycerides (2.05% *w*/*v*—Via Farma, São Paulo, Brazil), ethoxylated hydrogenated castor oil 40 (0.88% *w*/*v*—PharmaSpecial, São Paulo, Brazil), and Hy (0.05% *w*/*v*—Hangzhou APIChem Technology Co., Hangzhou, China). Dynamic light scattering (DLS) measurements and the electrophoretic mobility of the nanoparticles were then obtained using a pure NLC system (diluted in ultra-purified water 1:100 *v*/*v*, at a constant temperature of 25 °C at a scattering angle of 90°) as determined by the ZetaSizer Nano-ZS (Malvern Instruments, Malvern, UK). Finally, Hy was incorporated into the NLC and dispersed in HG at a 50:50% (*w*/*v*) ratio to obtain Hy.NLC-HG, which was then stored in amber bottles under refrigeration during the experiments. 

### 3.2. High-Resolution Scanning Electron Microscopy 

The pure HG and Hy.NLC-HG were lyophilized for 48 h, coated with a carbon conductive material (SEM-FEG, JEOL model JSM-7500F), and then analyzed to determine the average roughness values and interior structures of the hydrogels.

### 3.3. Mucoadhesion Strength Assay

For the mucoadhesion assay, bovine vaginal mucosa were soaked in artificial vaginal mucus, made according to modified methods [37,38], and attached to the base of a 10 mm diameter probe of a texture analyzer (TA-XT Plus Stable Micro Systems^®^, Surrey, UK). Falcon^®^ tubes holding either pure HG or Hy.NLC-HG were placed in a beaker of water, maintained at a consistent temperature of 37 ± 0.5 °C. To ensure contact between the sample surface and the vaginal mucosa, a downward force (0.05 N) was exerted for 60 s. The probe was then pulled upwards at a constant speed (1 mm.s^−1^) until the vaginal mucosa separated from it. The texture exponent software calculated the mucoadhesive strength (N) over time, with results represented as the mean and standard deviations from six replicates.

### 3.4. Determination of Syringability

A volume of 3 mL of pure HG and Hy.NLC-HG was loaded into a plastic syringe to a height of 3 mm to prevent air bubble formation. The syringe plunger was depressed at a constant speed (2 mm.s^−1^) over a 40 mm distance at 25 ± 0.5 °C [21]. The work required to extrude the sample from the syringe was determined from the area under the curve of the resultant force versus distance plot, using the mean and standard deviations from six replicates as determined by the texture analyzer (TA-XT Plus Stable Micro Systems^®^, Surrey, UK).

### 3.5. In Vitro Release Assay

The in vitro release assay was performed using Franz Diffusion Cells (Microette-Plus, Hanson Research Corporation^®^, Hanson, Chatsworth, CA, USA), comprising a 0.45 μm synthetic polysulfone membrane (Tuffryn^®^, Brazil) and a receiver solution composed of 0.01 M phosphate buffer (pH 5) and 3% Tween 80 at 37 °C ± 0.5 °C under 300 rpm stirring. A volume of 3000 μL of Hy solubilized in the receiver solution, as well as Hy.NLC-HG (each containing 500 μg.mL^−1^ of hypericin), was added to the Franz cell apparatus, with exposure to light avoided. Aliquots were collected at time intervals of 5, 15, 30, 60, 120, 240, 360, 480, and 720 min, then filtered using a polyether sulfone membrane filter (PES 0.22 μM). The amount of Hy released was determined using the HPLC method (Perkin Elmer liquid chromatograph with a UV–Vis detector at 590 nm) as per Kamal et al. [39], with: a C18 reverse phase column (Luna^®^ Phenomenex, Madrid, Spain, 250 mm × 4.6 mm, 5 μm), a flow rate of 1 mL.min^−1^, and acetonitrile:methanol:acetate buffer ammonium 10 mM (pH 5) used as the mobile phase at 54:36:10 (*v*/*v*/*v*) in an isocratic mode. The in vitro release profile was established by correlating time (minutes) versus Hy release (%) from the mean and standard deviation of triplicate measurements. 

### 3.6. Singlet Oxygen Generation Test

The Hy.NLC-HG/PDT solution (containing Hy at 500 μg.mL^−1^) in methanol was added to a standard solution of SOSG (Invitrogen) to achieve a final concentration of 2.5 μM. The intensity of SOSG fluorescence emission at 525 nm was determined by a spectrophotometer (Cary Eclipse, Agilent Technologies, UK) following the irradiation of the solution with white LED light (Fenix™, LD01, 113 J.cm^−2^), and in the absence of light, at 5 min intervals for a total of 15 min.

### 3.7. C. albicans Biofilm Inhibition Assay

For biofilm formation, 100 μL of a *C. albicans* ATCC 90028 suspension, adjusted according to the McFarland visual scale, was deposited into sterile 96-well microplates and incubated at 37 °C ± 0.5 °C for 2 h (Functionline CO_2_ incubator, Heraeus^®^, Hanau, Germany). Non-adherent *C. albicans* yeasts were removed from each well with sterile phosphate-buffered saline (PBS, Oxoid™). Then, 200 μL of yeast nitrogen base (YNB, Sigma Aldrich^®^, St. Louis, MO, USA) was added and the plates were incubated at 37 ± 0.5 °C for 48 h to facilitate mature biofilm formation. After the suspension was removed, the non-adherent biofilm cells were removed with PBS [40]. Biofilms not exposed to light and those exposed to LED light (Fenix ™, LD01, 113 J.cm^−2^ for 5 min after a 30-min pre-irradiation period) were treated with 100 μL of: (i) PBS/non-PDT (negative control), (ii) pure NLC/non-PDT, (iii) pure HG/non-PDT, (iv) free Hy/non-PDT (Hy solubilized in 20% DMSO from 1000 μg.mL^−1^), (v) free RB/non-PDT (Rose bengal, from 20 μM, positive contro, (vi) PBS/PDT, (vii) free Hy/PDT (from 1000 μg.mL^−1^), (viii) free RB/PDT (from 20 μM), (ix) Hy.NLC/PDT (from 1000 μg.mL^−1^), and (x) Hy.NLC-HG/PDT (from 1000 μg.mL^−1^). The detachment of the *C. albicans* biofilm was achieved by gently rubbing the bottom of the well with a pipette tip in a circular motion, vertically and horizontally, for 30 s [41]. Aliquots of 10 μL were spread over the surfaces of plates containing Sabouraud dextrose agar (SDA, Sigma Aldrich^®^), then incubated at 37 °C ± 0.5 °C for 24 h. The mean and standard deviation of the number of colony-forming units per milliliter (CFU.mL^−1^) were calculated from three replicates, and the data were transformed into log_10_. 

### 3.8. Antifungal Evaluation of the Systems in a VVC In Vivo Model

The in vivo experimental protocol was approved by the Ethics Committee on the Use of Animals (CEUA) of the School of Pharmaceutical Sciences, São Paulo State University, under protocol number 45/2018. Female mice of the C57BL/6 strain, aged 8 to 10 weeks and weighing approximately 20 to 25 g, were housed under controlled conditions of light and temperature. The cages were lined with sterilized shavings, and the mice had ad libitum access to food and water throughout the experimental period. According to the methodology adopted by Rodero et al. [40], five and two days before the fungal infection, the mice were placed in pseudoestrous conditions. This condition was maintained at two- and four-day intervals with a subcutaneous dose of 100 μL β-estradiol-solution (2 mg.mL^−1^) solubilized in sesame oil. On Day 0, each mouse was intravaginally inoculated with 20 μL of a *C. albicans* ATCC 10231 suspension at 2.5 × 10^8^ cells.mL^−1^ in sterile PBS. On Days 2, 4, 6, and 8 post-inoculation, vaginal lavages were performed in the vaginal cavities of the mice, using 70 μL of sterile PBS (pH 7.4). Four treatments were administered on Days 1, 3, 5, and 7 post-inoculation in the absence of light and under PDT, administering 20 μL of the samples intravaginally (*n* = 5/groups) twice a day. The following groups were evaluated: (i) inoculum/non-PDT (*C. albicans* control), (ii) vaginal cream/non-PDT (antifungal control based on benzoyl metronidazole, nystatin, and benzalkonium chloride), (iii) pure NLC/non-PDT, (iv) pure HG/non-PDT, (v) free Hy/non-PDT (500 μg.mL^−1^), and (vi) Hy.NLC-HG/non-PDT (500 μg.mL^−1^). For the PDT groups, after 30 min of pre-irradiation, the LED light (Fenix^TM^, LD01, 113 J.cm^−2^) was carefully applied for 5 min in the animals’ vaginal canal in two conditions: (vii) free Hy/PDT (500 μg.mL^−1^) and (viii) Hy.NLC-HG/PDT (500 μg.mL^−1^). All vaginal lavage fluid samples were subcultured onto plates containing SDA with chloramphenicol (SDA + Clo, Difco, San Diego, CA, USA) and incubated at 37 °C for 48 h (Microbiological Oven, Fanem). *C. albicans* counts (CFU) were determined and logarithmically transformed (log CFU). After eight days of the experiment, the mice were euthanized. The vaginal fluids of free Hy/PDT and Hy.NLC-HG/PDT were smeared on slides and stained with May–Grunwald Giemsa (MGG, Sigma Aldrich^®^, São Paulo, Brazil) solution (1:10). Vaginal *C. albicans* cell morphology was observed under optical microscopy at 40× magnification.

### 3.9. Statistical Analysis

Significant differences were considered for *p* < 0.05. The results of the syringeability, in vitro antibiofilm, and in vivo antifungal assays were compared for statistical significance using a one-way analysis of variance (ANOVA), followed by a post-hoc Tukey’s test. Mucoadhesion data were analyzed using a one-way ANOVA, followed by a post-hoc Dunnett’s multiple comparison test.

## 4. Conclusions

The hydrogels exhibited an exceptional mucoadhesiveness and extrusion capacity from a delivery device, which underscores their ease of administration and their capability to persist in the vaginal environment. The in vitro antifungal assay demonstrated that the integration of Hy into the hydrogel system did not alter the antifungal efficacy of Hy post-PDT. The PDT-mediated Hy.NLC-HG demonstrated a significant reduction in *C. albicans* biofilm growth. In the in vivo assay, groups treated with PDT displayed a predominance of yeast rather than hyphae, and Hy.NLC-HG showed a significant log level reduction in *C. albicans* colonies, thereby demonstrating its potential as a vaginal drug delivery system. Therefore, PDT-mediated NLCs dispersed in HG can be deemed a satisfactory approach to photodynamic therapy for controlling vaginal infections induced by *C. albicans*, such as VVC, and may also contribute to the pursuit of novel drug delivery systems.

## Data Availability

Data is contained within the article.
